# Sustainable Consumption: Will They Buy It Again? Factors Influencing the Intention to Repurchase Organic Food Grain

**DOI:** 10.3390/foods11193046

**Published:** 2022-09-30

**Authors:** Heena Thanki, Sweety Shah, Ankit Oza, Petrica Vizureanu, Dumitru Doru Burduhos-Nergis

**Affiliations:** 1 Department of Management, Shri Jairambhai Patel Institute of Business Management and Computer Applications (SJPI-NICM), Gandhinagar 382007, India; 2 Department of Management, L.J. Institute of Management Studies, L.J. University, Ahmedabad 382210, India; 3 Department of Computer Sciences and Engineering, Institute of Advanced Research, Gandhinagar 382426, India; 4 Faculty of Materials Science and Engineering, Gheorghe Asachi Technical University of Iasi, 700050 Iasi, Romania; 5 Technical Sciences Academy of Romania, Dacia Blvd 26, 030167 Bucharest, Romania

**Keywords:** organic grain, repurchase intention, Stimulus-Organism-Response (S-O-R) theory, theory of planned behaviour, consumer buying behaviour, repeat purchase

## Abstract

Global consumption trends point to rising demand for organic food as people become more health-conscious. The factors that people consider while making initial organic purchases have been discussed at length. However, the published research is scant about the factors that affect consumers’ propensity to repurchase organic goods. The present research fills this gap by focusing on what influences consumers’ decisions to repurchase organic grain. The Stimulus-Organism-Response (S-O-R) theory and the Theory of Planned Behaviour are the theoretical foundations of the present investigation. The consumer’s attitude toward organic grains and their desire to repurchase organic grains are influenced by health consciousness and previous experience. The repurchase intent was determined to be controlled by the buyer’s willingness to pay and their level of trust in the organic grain. This cross-sectional study collected the necessary data from five chosen urban centres in India. Smart PLS 3.2.9 was used to analyse the gathered data from 463 respondents. According to the findings, health consciousness and past experience favourably influence attitudes and repurchase intent. The trust that consumers have built up in organic grain as a result of past experiences is what drives their desire to make more purchases. Willingness to pay significantly controls and impacts the inclination to repurchase. The association between health consciousness and repurchase intention is partially mediated by attitude, as is the relationship between past experience and repurchase intention. The relationship between health consciousness and the desire to repurchase is partially mediated by the willingness to pay.

## 1. Introduction

The ongoing proliferation of capitalist society is manifested in environmental damage and the propagation of social and economic exploitation of individuals and societies [1]. After incurring much harm to the natural resources and environment, now we have started focusing on sustainable, green and ethical consumption. The phrase “sustainable consumption” is frequently used to refer to concerns such as human needs, equity, quality of life, resource efficiency, waste minimisation, life cycle thinking, consumer health and safety, consumer sovereignty and so forth [2]. Whereas ethical consumption is defined as “conscientious consumption that takes into account health, society and natural environment based on personal and moral beliefs” [3]. In this context, sustainable and ethical consumption is more or less represents the same issues and can be used interchangeably.

The major cause of climate change and environmental degradation is food consumption [4] and hence, the United Nations (UN) has designated sustainable consumption and production patterns as one of the key Sustainable Development Goals (SDGs) for achieving environmental sustainability. Agricultural and land use/land-use change activities account for one-third of worldwide anthropogenic GHG emissions, with agriculture and land use/land-use change activities contributing the most [5]. Many sustainability concerns have arisen as a result of current food production methods. They have an influence on pollution of the air, water and soil, biodiversity, ecosystems, energy consumption and climate change [6], as well as the environment and human health [7,8]. It is worth noting that organic farms maintain 30% more biodiversity than conventional farms, and they are more resistant to the effects of climate change such as drought and groundwater fluctuations [9,10].

In this light, the expansion of organic farming has the potential to prevent a significant amount of damage to nature and the surrounding environment. Nevertheless, transitioning to 100% organic farming is not a simple task, particularly for farmers, whose choice to do so is heavily influenced by the state of the organic product market [11,12]. Consequently, the expansion of, and demand for, organic products is a precondition for shifting agricultural production in more environmentally friendly and sustainable ways [13].

However, India is observing an astonishingly positive shift in consumption pattern toward organic products [14,15,16]. The COVID-19 event has served as a fuel that has further accelerated the demand for organic goods. Many customers’ perspectives have shifted as a result of the pandemic caused by the COVID19 outbreak. They are becoming more and more conscious of the dangers that come with abandoning the environment. Consumers now have a greater awareness of the health risks associated with hazardous chemically developed items, and as a result, they are choosing alternatives that are healthier. In India, the demand for organic products has risen steeply to an all-time high because of the COVID-19 epidemic, and sales of organic companies have increased by anywhere from 25% to 100% as a result.

Many studies have looked into the factors influencing the formation of organic product preferences and first-time/initial purchasing decisions in the Indian context [17,18,19,20,21,22,23,24]. These studies set the stage for the current investigation to identify the aspects that influence the repeat buying of organic grains specifically. In the context of consumer behaviour, “repurchase intention” states the likelihood that a customer will make a future buying of a product or service out of an ongoing desire to continue using and enjoying it. This emphasises the significance of investigating the factors influencing Indian consumers’ decisions to repurchase organic grains. Thus, it is imperative to study the key drivers of the repurchase intention of organic grain consumers in the Indian context. Second, the study adds to the body of knowledge by incorporating theories of behaviour and ethics. The study also analyses the mediating impact of (i) willingness to pay between health consciousness and repurchase intention, (ii) attitude between health consciousness and repurchase intention, (iii) attitude between experience and repurchase intention and (iv) trust between experience and repurchase intention. Lastly, this research elucidates the factors affecting organic grain purchase behaviour by including many variables.

## 2. Literature Review and Hypotheses Development

### 2.1. Theoretical Background

The consumption of harmful chemicals, such as pesticide and agrochemicals, is a growing concern among consumers. Organic food purchasers are influenced by both individual and ecological considerations. Organic food has become popular recently because of people’s concerns about chemicals and their impact on their health [24,25,26,27,28,29,30,31]. While many different psychological models are proposed to envisage individuals’ ecologically appropriate behaviour, the TPB model developed by Ajzen [32] has gained considerable traction due to its significant explanatory power. It is an extension of the theory of reasoned action given by Fishbein and Ajzen [33] and helps the study of human behaviour in many different contexts, such as health [34,35] and nutrition [36,37,38,39,40,41]. Further, as per the S-O-R framework developed by Mehrabian and Russell [42] “external environmental act as stimuli (S) to individuals’ internal states/evaluations organism (O), which drives their behavioural responses (R).” For the current study, the attitude is taken from TPB theory [32] and extended by adding willingness to purchase, which is an antecedent of controls beliefs and known as perceived behavioural control in the TPB model, past experience from extended TPB Model by East [43], health consciousness (people will show the behaviour if it is personally benefiting them) extracted from “The Methods of Ethics” presented by Sidgwick [44], and trust from customer trust model by Morgan and Hunt [45]. Similar to how the external image of organic food’s capability and social responsibility (stimuli) leads to an internal state/evaluation of consumer trust (organism) that drives consumers’ intention to repurchase (response). Along with this, the mediation effects have been studied among the constructs to understand the interlinkages of the all-independent variables selected for the research.

### 2.2. Hypotheses Development

#### 2.2.1. Health Consciousness

The number of health-conscious consumers influences organic food repurchase intention. “Health consciousness” is consumers’ “readiness to identify with and act on health” [46]. Many people believe organic food is better than conventional because of its health benefits [47] as it is grown without the use of any synthetic chemicals or genetic engineering [46,48]. As more people realise how organic foods are made, they become a healthier alternative [49,50,51,52] and they are most likely to buy organic foods [28]. Health concerns are a direct cause of a person’s positive attitude toward organic products and propensity to buy them [46,53,54,55,56]. Health-conscious buyers will pay more for organic items [57] for strong nutritional value or natural content [58]. This increases the customer’s desire to buy and readiness to pay. Given this, the hypotheses are presented as:

**H1** **(a).** *Health consciousness has a positive and substantial effect on the willingness to pay for organic food grains.*

**H1** **(b).** *Health consciousness has a positive and substantial effect on the attitude toward organic food grains.*

**H1** **(c).** *Health consciousness has a positive and substantial effect on the repurchase intention of organic food grains.*

#### 2.2.2. Past Experience

The positive experiences that customers have with a company’s products or services create emotional value [59]. In today’s experience economy, where consumers expect to be favourably and emotionally influenced at every level, the product must create a unique and psychological experience for each customer. Past experience is the foundation for future decision-making [60]. Positive associations with a brand or product are associated with increased brand loyalty and generate repurchase, according to research [61]. Consumers place a premium on positive brand experiences [62,63]. A customer’s willingness to make a repeat purchase is influenced by factors such as their attitude toward the product’s value, quality and price. Furthermore, studies have shown that one’s experience may have a secondary impact on their willingness to buy organic foods [55,64]. Past experience significantly impacts the development of customer loyalty and trust, and it tends to increase future transaction intentions. This leads to the following hypothesis:

**H2** **(a).** *Past experience has a positive and substantial effect on the attitude towards the organic food grains.*

**H2** **(b).** *Past experience has a positive and substantial effect on the trust for the organic food grains.*

**H2** **(c).** *Past experience has a positive and substantial effect on the repurchase intention of organic food grains.*

#### 2.2.3. Willingness to Pay

The term “willingness to pay” is used to describe the highest possible purchase price for a good or service [65]. Organic grains are more expensive than conventional grains [66], and the value difference between the two is high. Consumers frequently cite the greater cost of organic products as a reason for their reluctance to purchase them [56,67,68,69]. However, because the price is a critical factor in organic food consumer behaviour [70], it has been extensively studied for organic products. Despite the common belief among consumers that organic products is more costly, Massey et al. [71] found that “intention to purchase remains high.” This may be because organic product buyers are less price conscious than those who do not [72]. The more a buyer is ready to pay a premium for organic grains, the less negative impact its price has and the more often they buy it [73]. Repurchases of a quality product, such organic grains with health and environmental benefits [74,75], prepare consumers to pay more for prospective benefits [76]. Thus, it leads us to propose the following hypothesis:

**H3** **(a).** *Willingness to pay has a positive and substantial effect on the repurchase intention of organic food grains.*

**H3** **(b).** *Willingness to pay mediates health consciousness and the repurchase intention of organic food grains.*

#### 2.2.4. Attitude

One definition of attitude is a consumer’s propensity for or aversion to a given action [64]. A person’s attitude can be understood as their willingness to engage in or abstain from a specific behaviour [39], and this action leads to behavioural or user intentions [33]. Consumers’ attitude toward organic food is a significant factor in determining their purchase behaviour [77]. Consumers with a strong sense of morality and an understanding of the environmental impact are likely to be interested in purchasing organic choices [41,78,79]. There is a positive result due to attitude and use of organic products [18]. An increase in positive attitudes toward organic food has been linked to greater intent to buy [56,80]. Additionally, it was found that consumers’ hedonic attitudes influenced their preference for organic food due to attributes like organic food’s nutrition and natural content [81]. In light of this, we put forward the following hypothesis:

**H4** **(a).** *Attitude has a positive and substantial effect on the repurchase intention of organic food grains.*

**H4** **(b).** *Attitude mediates past experience and the repurchase intention of organic food grains.*

**H4** **(c).** *Attitude mediates health consciousness and the repurchase intention of organic food grains.*

#### 2.2.5. Trust

The term “trust” is used to describe an individual’s or group’s conviction that another entity can be relied upon to provide the desired result [82]. As Moorman, Deshpande and Zaltman [83] put it, trust is the behavioural intention underlying “willingness”. Customers place their faith on a company when they have confidence in its consistently delivering high-quality service [84]. Trust from customers is a critical factor in maintaining repeat business and attracting new ones. Previous research has found that trust influences consumer behaviour [85,86,87,88,89]. A growing research literature shows that consumer trust is a significant factor in their decision to consume and purchase organic food products [89,90,91,92]. Given this, the hypothesis is presented as:

**H5** **(a).** *Trust has a positive and substantial effect on the repurchase intention of organic food grains.*

**H5** **(b).** *Trust mediates past experience and the repurchase intention of organic food grains.*

## 3. Materials and Methods

We set out to answer three central questions with our research: (1) What influences consumers’ decisions to repurchase organic food grains; (2) how willingness to pay and attitude mediate the relationship between health consciousness and repurchase intention of organic food grains; (3) how attitude and trust mediate the relationship between past experience and repurchase intention of organic food grains.

**Figure 1:**
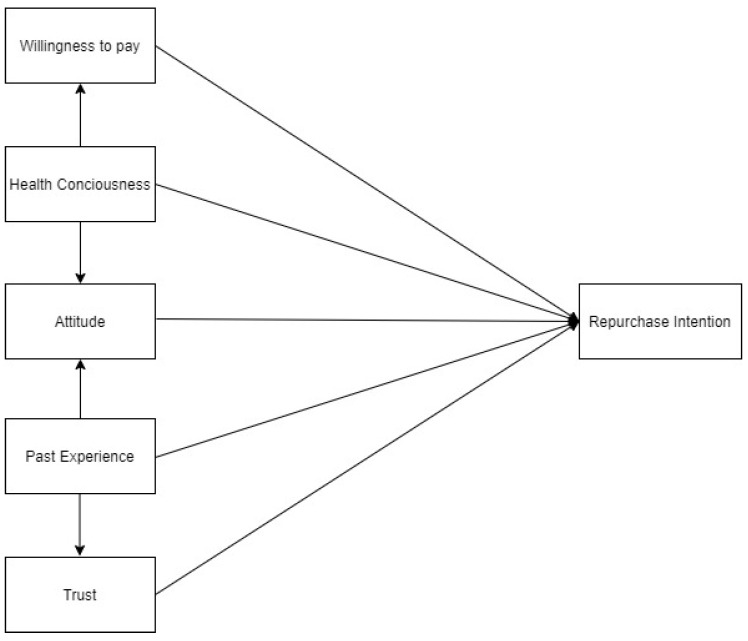
Conceptual model.

The quantitative method was applied to examine the hypotheses summarised in Figure 1.

### 3.1. Measures

The data for the study were gathered using a structured questionnaire. The instrument had three sections, the first of which collected respondents’ basic demographic details, including age, gender, marital status, education level, occupation and annual income. The other section asked respondents to rate the impact of various factors on their repurchase intention for organic grains, such as willingness to pay, health consciousness, attitude, past experience and trust. In the final segment, respondents’ willingness to repurchase organic food grains was assessed using Likert scale questions. The respondents rated their agreement with each statement from 1 (completely disagree) to 5 (completely agree).

The TPB developed by Ajzen [32] measure was used in the study to assess the attitude and repurchase intention of consumers for organic food grains. For health consciousness, the scale was developed from Basha and Lal [93] and willingness to pay from Molinillo et al. [94], past experience from Huang [95] and trust from Sultan et al. [96].

### 3.2. Population and Sampling

The study population for this study was organic food consumers. There is already a burgeoning organic food market in India’s major urban centres. The information was gathered in cities with high-income households, such as Mumbai, Ahmedabad, Hyderabad, Pune and New Delhi (NCR). First, we used the snowball sampling technique to reach out to 552 consumers of organic food grain through in-person visits and emails. The respondents were assured of the confidentiality of the data. However, 89 responses out of 552 were excluded as they were incomplete. Thus, 463 responses were processed for further data analysis (see Table 1). The descriptive statistics showed that 124 (26.78%), 114 (24.63%), 59 (12.73%), 87 (17.79%) and 79 (17.06%) were from Mumbai, New Delhi (NCR), Ahmedabad, Hyderabad and Pune respectively. The total respondents include 112 males (24.19%) and 351 females (75.81%). The descriptive analysis also reflects that 81 (17.42%), 147 (31.75%), 136 (29.37%), 85 (18.36%) and 14 (3.02%) respondents were of 26–35 years, 36–45 years, 46–55 years, 56–65 years and 65 years and above. Moreover, 389 (84.02%) respondents were married, and 73 (15.98%) were unmarried. Data represent undergraduate 28 (6.04%), graduate 171 (36.93%), postgraduate professional 116 (25.05%), doctorate 37 (7.99%) and others 11 (2.37%). Data include 124 (26.78%) housewives, 93 (20.09%) salaried—government sector employees, 165 (35.64%) salaried—private sector employees, 49 (10.58%) self-employed and 32 (6.91%) business people. Finally, the household income between ₹ 250k and ₹ 400k, ₹ 400k and ₹ 650k, ₹ 650k and ₹ 800k, ₹ 800k and ₹ 1000k and above ₹ 1000k were 2 (0.431%), 55 (11.88%), 98 (21.17%), 156 (33.69%) and 152 (32.83%).

### 3.3. Data Analysis

Partial least square equation modelling (PLS-SEM) was used to analyse the data in this study with the help of the statistical tool Smart PLS (3.2.9). This method has become increasingly popular in the literature on human resource management, marketing and related topics [97,98]. PLS-SEM is used to forecast the effects of independent variables on dependent variables [98]. Davari and Rezazadeh [99] made a similar point, arguing that this method works well for predicting multiple equations in the research model and establishing causality between variables. Given its ability to examine the inherently difficult-to-examine and unobservable latent constructs, SEM is widely regarded as the best method for measuring direct and indirect paths [100]. Consequently, this method is selected for the current study.

## 4. Results

### 4.1. Measurement Model

The present study examined the measurement model approach to assess the CA, CR and AVE. Table 2 displays the correlation between CA and CR in terms of attitude (0.759, 0.844), health awareness (0.802, 0.883), past experience (0.921, 0.944), trust (0.939, 0.951), willingness to pay (0.753, 0.845) and repurchase intention (0.870, 0.921). This study confirms that the CA and CR values are within a reasonable range (above 0.70), as recommended by Hair, Ringle and Sarstedt [98]. To examine discriminant validity, we calculated the “Fornell–Larcker” and “Heterotrait–Monotrait (HTMT)” ratios [101]. Table 3 displays the results of tests conducted as per Fornell and Larcker, where the values are greater than the correlations between the variables. Recent studies have shown that the HTMT ratio is superior to Fornell and Larcker [102] (see Table 4). The ratios obtained using HTMT are under the minimum allowable values of 0.090. In addition, we looked at AVE values and outer factor loadings to test the convergent validity, and all the AVE values were above the 0.50 threshold (attitude: 0.577, health consciousness: 0.717, experience: 0.808, trust: 0.734, willingness to pay: 0.585, and repurchase intention: 0.794), as recommended by Henseler, Hubona and Ray [102] (see Table 2). At the screening of items, one items had factor loading below 0.7, so it was removed from the analysis. To investigate the CMB in PLS-SEM, Kock [103] recommends variance inflated factors (VIF) test. VIF values in this study are within the range suggested by Hair et al. [100], indicating no multicollinearity problem with the data (see Table 5).

### 4.2. Assessment of Structural Model

**Figure 2:**
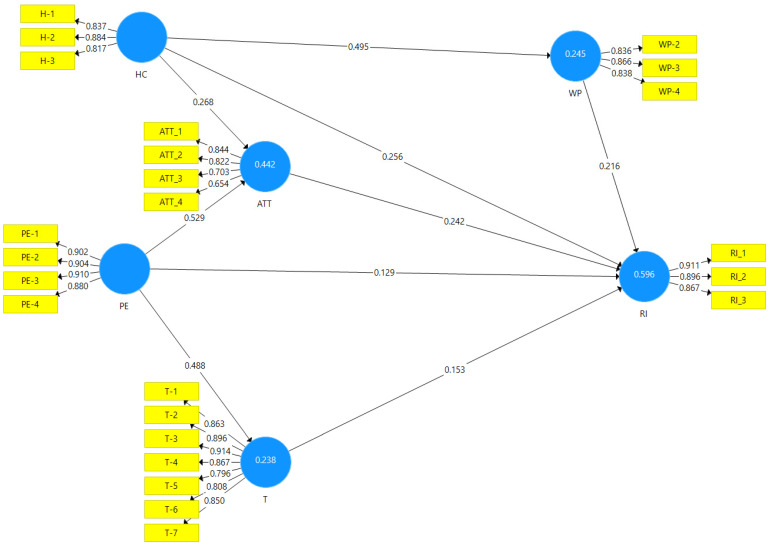
Run model.

To evaluate the structure equation model, we used 5000 bootstraps in the Smart PLS software. Standardised root mean square (SRMR) values below 0.08 are recommended by Henseler, Hubona and Ray [102] and Cho et al. [104]. As a result, this study has a significant model fit (*p* = 0.058) (see Table 6). Coefficient of determination (R^2^) values should be greater than 0.1 [105]. The current research analysed that 44% variance occurred in attitude, explained by health consciousness and past experience; 24.5% variance happened in willingness to pay, explained by health consciousness, 23.8% variance occurred in trust, explained by past experience and 59.6% variance occurred in repurchase intention explained by a willingness to pay, health consciousness, attitude, past experience and trust (see Figure 2 and Table 6). Further, Q^2^ must have a value above zero. Thus, the results of this study were consistent with the significance level, and the predictive relevance of the study model was attained (see Table 6) [106].

The PLS-SEM results indicate that health consciousness positively impacts willingness to pay (β = 0.495, t > 1.96) and attitude (β = 0.268, t > 1.96). Health consciousness (β = 0.256, t > 1.96) also has positive impact on repurchase intention, thus H1 (a), (b) and (c) are accepted. Past experience has significant positive impact on attitude (β = 0.529, t > 1.96), trust (β = 0.488, t > 1.96), and has repurchase intention (β = 0.129, t > 1.96) hence, hypotheses H2 (a), (b) and (c) are accepted. Willingness to pay had a substantial positive effect on repurchase intent (β = 0.216, t > 1.96) and supported H3 (a). Attitude ((β = 0.242, t > 1.96) positively impacts repurchase intention so H4 (a) is accepted. Lastly, trust (β = 0.153, t > 1.96) significantly positively affect the repurchase intention of organic food grains so H5 (a) is accepted (see Table 6).

### 4.3. Mediation Analysis

“Mediation” refers to an indirect effect that may help determine the association between selected variables [107]. This study used a mediation analysis test to investigate- (i) the mediating role of attitude between health consciousness and repurchase intention of organic grains; (ii) the mediating role of attitude between past experience and repurchase intention of organic grains; (iii) the mediating role of trust between past experience and repurchase intention of organic grains and (iv) the mediating role of willingness to purchase between health consciousness and repurchase intention of organic grains. The mediation effect can be examined by using the bootstrapping approach [108].

The mediation analysis was carried out by computing total indirect effects and specific indirect effects. Table 7 presents the results of the investigation on the use of mediation.

The results in the above tables show that willingness to pay is mediating the relationship of health consciousness and repurchase intention (*p* = 0.000, t statistics = 3.684) thus H3 (b) is accepted. Attitude mediates significantly between past experience and repurchase intention (*p* = 0.000, t statistics = 5.095) and between health consciousness and repurchase intention (*p* = 0.000, t statistics = 4.267), so hypotheses H4 (b) and H4 (c) are accepted. Similarly, trust is mediating the relationship between past experience and repurchase intention (*p* = 0.001, t statistics= 3.381); and hypothesis H5 (b) is accepted.

## 5. Discussion

The current research addresses several research gaps by examining the repurchase intent of organic food grain and how consumers behave while making these decisions. The proposed model first delves into the underexplored question of what motivates consumers to repurchase organic products. Previous studies have shown that a significant portion of consumers is put off from purchasing organic products due to the higher prices. According to the findings of the most recent study, the level of a consumer’s willingness to pay a premium predicts the level of the consumer’s frequency of making purchases. The results of this inquiry are therefore consistent with those of earlier studies [72,73]. Consumers’ awareness of the need of maintaining their health is considered as a catalyst that favourably influences their propensity to spend. In addition, one’s willingness to pay for the products is a factor that plays a role as a mediator in the interaction that takes place between health consciousness and the intention to repurchase the product again. These findings are in line with those reported in various other areas of study [46,48,53,54,55,56] and indicate that health awareness has a substantial and favourable impact on consumers’ attitudes, as well as their willingness to spend and propensity to make repeat purchases. The consumers’ level of health consciousness is the most critical element determining whether or not they intend to buy organic grain. Because of their view that organic grains may greatly improve health, customers who are health concerned have a more optimistic outlook on organic grains and are willing to spend more money on organic products because of this conviction.

The likelihood of repeat purchases of a product is found to increase after a favourable experience that is also pleasant and entertaining; this conclusion is in accordance with a similar finding made by Nalchy, Rasoulian and Boojari [61]. The consumer is more likely to have a favourable attitude toward the product and a higher level of trust in the product if they have had a pleasant experience in the past with the product; this conclusion validates the findings reported in [55,64,80]. When a consumer uses a product for the first time, their perspective on the product will be revaluated, and as a consequence of this review, the client’s attitude toward the product will shift. When a person has a positive, delightful and pleasurable experience, they are more likely to have a positive attitude towards the product; the same is true when they have a negative attitude towards the product. When a person has a positive attitude towards the product, they are more likely to have a positive attitude towards the product. An individual’s inclination to repurchase a product is influenced by the consumer’s trust and confidence in the product [90,91,92,93,109], both of which are bolstered by a positive prior experience with the product.

## 6. Implications

Consumer research on organic food grains as part of the agri-foods industry has lagged far behind. This investigation deepens the knowledge of consumer preferences for organic food grains by elaborating on the S-O-R and TPB. When it comes to the TPB model, this study has also included the extended TPB model, egoism theory of ethics and customer trust model to understand the repurchase intention of consumers. The tested model can also be used for other instances, even though the research context is organic food grains. Even if actual consumption patterns are not measured, the study’s consideration of consumers’ intentions regarding organic agri-food may make the results more applicable. Adding a fresh viewpoint to the agri-food literature, the current research offers a signal to aid manufacturers and dealers in enticing customers. More than that, it offers theoretical justifications for how to react to consumers repurchase decisions. As health consciousness has been shown to influence consumers’ decisions to repurchase organic food grains largely, this research suggests that producers and marketers of organic food grains should conduct educational marketing campaigns about the health benefits of such products among households. They can advertise the organic products with their health benefits targeting all age groups to attract first-time consumers and induce repurchase. The interest in nutritional foods among the younger generations has been growing recently, and the COVID-19 pandemic has turned consumers towards a healthy lifestyle. The campaigning needs more planning and coordination to portray the right message, both in terms of educating the public and drawing attention to the unique advantages of repurchasing organic grains. The marketers make it available in general trade and focus on the modern trade supply chain to save time and energy for consumers in reaching such food items, specifically in a country like India. The Indian Government may consider improving organic farming by creating more proactive policies that encourage the practice and deflecting subsidies to support organic farming. This would help remove supply-related barriers.

## 7. Future Scope and Limitations

Here the study has considered constructs from various behavioural studies; further, it can be extended with more emotional variables affecting the repurchase intention. The study has covered five urban centres of the country; further research should be covered by adding all urban cities. There is an evident lack of data for producers, marketers and the federal government to make strategic decisions regarding the growth of the organic food grain market, and this study shows that more research needs to be done in this area. This study has some limitations, including small sample size. Given that our sample was limited to urban centres of India and that a variety of variables affect people’s behaviour and action, the results of this study should not be extrapolated beyond the selected sample. The sample for the current study was from middle income or higher income group, if in future the lower income group is to be studied, price can also be explored as a inhibiting factor. Moreover, while the results do lend credence to the underlying theory, the use of a self-reported survey raises concerns about the study’s external validity.

## Figures and Tables

**Table 1:** Descriptive statistics.

Variables	Category	Frequency	Percent
Location	Mumbai	124	26.78
	New Delhi (NCR)	114	24.62
	Ahmedabad	59	12.73
	Hyderabad	87	18.79
	Pune	79	17.06
Gender	Male	112	24.19
	Female	351	75.81
Age	26–35	81	17.42
	36–45	147	31.75
	46–55	136	29.37
	56–65	85	18.36
	65 and above	14	3.02
Marital status	Married	389	84.02
	Unmarried	73	15.98
Education Qualification	Undergraduate	28	6.04
	Graduate	171	36.93
	Postgraduate professional	116	25.05
	Doctorate	37	7.99
	Other	11	2.37
Employment	Housewife	124	26.78
	Salaried—government sector employee	93	20.09
	Salaried—private sector employee	165	35.64
	Self-employed	49	10.58
	Business	32	6.91
Household Annual Income	Between ₹ 250k and ₹ 400k	2	0.431
	Between ₹ 400k and ₹ 650k	55	11.88
	Between ₹ 650k and ₹ 800k	98	21.17
	Between ₹ 800k and ₹ 1000k	156	33.69
	Above ₹ 1000k	152	32.83

(Source: authors’ calculation using SPSS).

**Table 2:** Reliability and validity.

	Item Code	Loading	Outer Weights	CA	CR	AVE
Attitude (ATT)				0.759	0.844	0.577
	ATT_1	0.844	0.378			
	ATT_2	0.822	0.392			
	ATT_3	0.703	0.265			
	ATT_4	0.754	0.263			
Health Consciousness (HC)				0.802	0.883	0.717
	HC_1	0.837	0.388			
	HC_2	0.884	0.386			
	HC_3	0.817	0.409			
Past Experience (PE)				0.921	0.944	0.808
	PE_1	0.902	0.284			
	PE_2	0.904	0.274			
	PE_3	0.910	0.269			
	PE_4	0.880	0.286			
Trust (T)				0.939	0.951	0.734
	T_1	0.863	0.174			
	T_2	0.896	0.157			
	T_3	0.914	0.182			
	T_4	0.867	0.159			
	T_5	0.796	0.182			
	T_6	0.808	0.149			
	T_7	0.850	0.165			
Willingness to Pay (WP)				0.753	0.845	0.585
	WP_2	0.836	0.405			
	WP_3	0.866	0.367			
	WP_4	0.838	0.409			
Repurchase Intention (RI)				0.87	0.921	0.794
	RI_1	0.911	0.378			
	RI_2	0.896	0.379			
	RI_3	0.867	0.365			

Source: Authors’ calculations conducted using Smart PLS 3.2.9. (Note: “average variance extracted (AVE)”; “Cronbach’s alpha (CA)”; “composite reliability (CR)”).

**Table 3:** Fornell–Larcker criterion.

	ATT	HC	PE	RI	T	WP
ATT	0.760					
HC	0.436	0.847				
PE	0.614	0.317	0.899			
RI	0.637	0.577	0.535	0.891		
T	0.548	0.440	0.488	0.587	0.857	
WP	0.557	0.495	0.472	0.627	0.581	0.847

Source: Authors’ calculations conducted using Smart PLS 3.2.9.

**Table 4:** Heterotrait–Monotrait ratio (HTMT).

	ATT	H	PE	RI	T	WP
ATT						
H	0.547					
PE	0.712	0.369				
RI	0.768	0.69	0.597			
T	0.646	0.507	0.523	0.645		
WP	0.68	0.664	0.527	0.743	0.628	

Source: Authors’ calculations conducted using Smart PLS 3.2.9.

**Table 5:** Inner VIF Values.

Independent Variables	WP	ATT	T	RI
ATT				2.022
H	1	1.112		1.465
PE		1.112	1	1.718
T				1.748
WP				1.831

Source: Authors’ calculations were conducted using Smart PLS 3.2.9.

**Table 6:** Path coefficients and fitness indices for the structural model.

Hypothesis Number	Hypothesis	β (Path Coefficient)	T Statistics (|O/STDEV|)	R^2^	Q^2^	SRMR	RSM Theta	Result
H1 (a)	HC -> WP	0.495	12.949	0.245	0.171	0.058	0.113	Accepted
H1 (b)	HC -> ATT	0.268	6.863	0.442	0.247			Accepted
H2 (a)	PE -> ATT	0.529	12.974					Accepted
H2 (b)	PE -> T	0.488	11.574	0.238	0.171			Accepted
H1 (c)	HC -> RI	0.256	6.358	0.596	0.466			Accepted
H2 (c)	PE -> RI	0.129	3.127					Accepted
H3 (a)	WP -> RI	0.216	3.971					Accepted
H4 (a)	ATT -> RI	0.242	5.476					Accepted
H5 (a)	T -> RI	0.153	3.567					Accepted

Source: Authors’ calculations conducted using Smart PLS 3.2.9.

**Table 7:** Specific indirect effects.

		β (Path Coefficient)	Sample Mean (M)	Standard Deviation (STDEV)	T Statistics (|O/STDEV|)	*p*-Values	Result
H3 (b)	HC -> WP -> RI	0.107	0.108	0.029	3.684	0.000	Accepted
H4 (b)	HC -> ATT_ -> RI	0.065	0.065	0.015	4.267	0.000	Accepted
H4 (c)	PE -> ATT_ -> RI	0.128	0.128	0.025	5.095	0.000	Accepted
H5 (b)	PE -> T -> RI	0.075	0.074	0.022	3.381	0.001	Accepted

Source: Authors’ calculations conducted using Smart PLS 3.2.9.

## Data Availability

Data is contained within the article.
